# Investigation of emission plane control in GaInN/GaN multiple-quantum shells for efficient nanowire-based LEDs

**DOI:** 10.1039/d3na01101a

**Published:** 2024-03-27

**Authors:** Soma Inaba, Weifang Lu, Ayaka Shima, Shiori Ii, Mizuki Takahashi, Yuki Yamanaka, Yuta Hattori, Kosei Kubota, Kai Huang, Motoaki Iwaya, Tetsuya Takeuchi, Satoshi Kamiyama

**Affiliations:** a Department of Materials Science and Engineering, Meijo University 1-501 Shiogamaguchi, Tenpaku-ku Nagoya 468-8502 Japan; b Fujian Key Laboratory of Semiconductor Materials and Applications, CI Center for OSED, Department of Physics, Xiamen University Xiamen 361005 China weilu.weif@xmu.edu.cn; c Future Display Institute in Xiamen Xiamen 361005 China

## Abstract

Significant attention has been directed toward core–shell GaInN/GaN multiple-quantum shell (MQS) nanowires (NWs) in the context of high-efficiency micro light-emitting diodes (micro-LEDs). These independent three-dimensional NWs offer the advantage of reducing the impact of sidewall etching regions. Furthermore, the emitting plane on the sidewalls demonstrates either nonpolar or semipolar orientation, while the dislocation density is exceptionally low. In this study, we assessed how changes in the NW morphology are affected by GaInN/GaN superlattice (SL) structures grown at varying growth temperatures, as well as control of the emission plane *via* the p-GaN shell and emission sizes. The SL growth rate was enhanced at elevated growth temperatures, accompanied by the shrinkage of the (0001)-plane and expansion of the (11̄01)-plane on the NWs. The samples exhibited a higher light output when the SLs were grown at elevated temperatures compared to those grown with lower temperatures. A similar trend was observed for the samples with a gradual temperature transition during the growth. These findings indicate that the dimensions of the (0001)-plane can be controlled through SL growth, which in turn influences the emission properties of NW-LEDs. In addition, the emission properties of NW-LEDs with different growth time p-GaN shells and different emission sizes were investigated. Based on the NW-LED characteristics, it was revealed that the weak emission of the (0001)-plane was the dominant factor for the limited light output, and the most effective way to realize high efficiency devices is to suppress current injection into the apex or minimize the grown (0001)-plane region. Overall, it is one promising way to control the emission planes of NWs, which holds significant relevance for the potential application of NW-LEDs in the realm of micro-LEDs.

## Abstract 2

To improve the emission properties of multiple-quantum-shells nanowire-based LEDs, we assessed the effect of GaInN/GaN superlattices grown at varying growth temperatures, as well as the control of emission plane *via* p-GaN shell and emission sizes.

## Graphical abstract

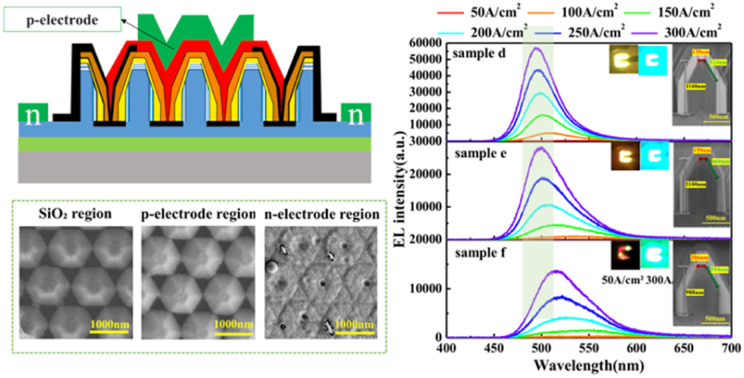

## 1. Introduction

Micro light-emitting diodes (micro-LEDs), based on group III nitride semiconductors, have garnered significant attention as promising candidates for next-generation displays. This popularity stems from their exceptional traits, including high contrast, fast response, broad wavelength range, and low power consumption.^1–6^ Nevertheless, there are notable challenges that persist in improving the efficiency of micro-LEDs. The foremost challenge is the efficiency drop observed at longer wavelengths. Achieving emission in these long-wavelengths necessitates an increase in the InN mole fraction within the active layer. However, elevating the InN mole fraction usually amplifies the lattice mismatch between GaN and InGaN, which can exacerbate the Quantum-Confined Stark Effect (QCSE) due to the growing piezoelectric field. In turn, such a phenomenon leads to a decline in luminous efficiency.^7,8^ The second challenge revolves around the degradation of the external quantum efficiency (EQE), which arises from elevated Shockley–Read–Hall non-radiative recombination rates attributable to defects and damage to the mesa sidewalls, stemming from dry etching with plasma.^9,10^ This degradation trend becomes more pronounced with decreasing device size, as the ratio of the sidewalls to the mesa's top surface increases, consequently exacerbating the EQE decline.^11–13^ Although approaches like applying passivation films onto the sidewalls have demonstrated a certain degree of effectiveness in mitigating defects and damage caused by dry etching,^14^ this predicament remains inadequately resolved according to the most recent advances in the field. A promising strategy to address the aforementioned challenges involves the utilization of LEDs employing coaxial GaInN/GaN multiple-quantum shell (MQS) nanowires (NWs), which have garnered significant attention. These GaInN/GaN NWs exhibit a distinctive three-dimensional growth pattern, forming hexagonal prismatic microcrystals characterized by an impressively low density of dislocation defects.^15–19^ This unique structural configuration enables NW-LEDs to utilize non-polar (101̄0) or semi-polar (11̄01)-plane surfaces as active layers, resulting in a reduction of the QCSE effect to either 0 or 1/3 of its magnitude.^20^ Fine-tuning factors such as the pitch, diameter, and height of the NWs, as well as modulating In incorporation, allows precise control over the emission wavelength of NW-LEDs. This level of control is achievable due to the manageable In desorption influenced by the growth rate of the NWs.^21–23^ Specifically, the (11̄01)-plane of NWs holds promise for highly efficient emission in the long-wavelength spectrum. This is due to the potential reduction of QCSE to one-third or less, coupled with reported higher level of InN incorporation compared to other planes.^24^ Notably, NW-LEDs are being actively explored for their potential to realize micro-LEDs, thus presenting a pathway toward high impact in this field.

In the fabrication process of micro-LEDs utilizing core–shell NWs, the region of the mesa sidewall exposed through dry etching can be minimized by selectively removing NWs located adjacent to the mesa's edge area. Furthermore, owing to the unique structure of each individual NW, it is feasible to devise the device configuration in a manner that prevents the injection of current into NWs that may have been compromised by the dry etching process, particularly those situated at the outermost periphery. As a result, the periodic arrangement of NWs and the utilization of a core–shell structure hold significant promise as an approach towards achieving high-efficiency micro-LEDs from multiple perspectives, including the potential to mitigate efficiency degradation in terms of EQE. Nevertheless, the high growth temperatures and low V/III ratios in n-GaN core growth of NW-LEDs may result in low crystal quality MQS due to the elevated density of point defects.^25^ As a result, NW-LEDs still struggle with achieving a comparable luminous efficiency when compared to their planar LED counterparts. Specifically, the MQS on the (0001)-plane exhibits reduced emission efficiency due to the serrated rough surface caused by the heightened InN mole fraction, coupled with the influence of point defects.^16,26,27^ To ameliorate these issues, the integration of superlattice (SL) structures offers a potential solution to suppress point defects, while the luminescent surface can also be controlled by NW morphology and current density. In the case of planar (0001)-plane LEDs, the introduction of SLs has demonstrated improvement in emission properties,^28,29^ which enhances the quality of the active layer by capturing point defects that tend to migrate toward the active region during SL growth.^30^ For NW-LEDs, our research laboratory has also reported a 3.1-fold enhancement in electroluminescence (EL) intensity consequent to the introduction of SLs.^31^ This was ascribed to the effect of the SL in suppressing the propagation of point defects, alongside alteration in the emitting plane due to the modifications in the shape of the NWs prompted by the incorporation of the SL. Additionally, there have been reports indicating that a higher InN mole fraction within GaInN/GaN SLs promotes the entrapment of point defects.^32,33^ Regarding planar LEDs, several studies have explored the growth temperature of SLs,^34,35^ which has not been reported in NW-LEDs.

In this work, we focused on a comprehensive investigation of the morphology and emission properties of NW-LEDs with varying growth temperatures of GaInN/GaN SLs. Detailed morphology features were characterized by scanning electron microscopy (SEM, SU5000, Hitachi High-Technologies Corporation, Minato-ku, Tokyo, Japan), and device performances were assessed using EL and current density–voltage–light output (*J*–*V*–*L*) measurements. Changing the growth temperature of the SL led to noticeable alterations in the NW morphology, especially at a high temperature. Consequently, the MQS of the (0001)-plane region was enlarged and the optical light output decreased for the sample with low-temperature-grown SLs. Evidently, the growth of SLs played a pivotal role in controlling the size of the (0001)-plane region. Since the emission of this specific region was identified to substantially impact the overall emission of the NWs, we delved into exploring the relationship among injection current density, changes in the p-GaN shell and emission area of devices. Overall, the findings derived from this work offer valuable insights into the shape control of NWs by the SL and emission features with different p-GaN shell growth and NW-LED device size.

## 2. Experimental section

**Fig. 1:**
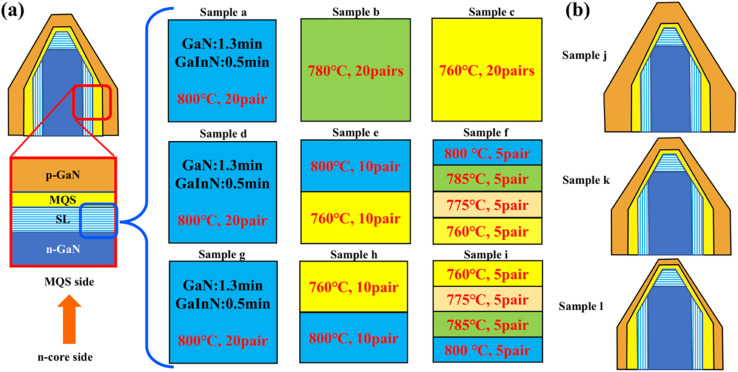
(a) Schematic diagrams of the NW samples with GaInN/GaN SLs. The detailed structures on the (101̄0)-plane are illustrated from the n-core side to the p-GaN shell. The detailed growth conditions of SLs in three different batches of NW samples are shown in the left, while the bottom represents the n-core side and the upper side represents the MQS side. (b) Schematic diagrams of the NW samples j, k, and l with different p-GaN growth times.

NW samples for device fabrication were synthesized using a selective growth method *via* a metal–organic chemical vapor deposition (MOCVD) system (SR 2000, TAIYO NIPPON SANSO Co., Shinagawa-ku, Tokyo, Japan) on n-GaN/sapphire templates.^16^ The templates patterned with a hole diameter of 320 nm and a pitch of 1200 nm were prepared by nanoimprint lithography and inductively coupled plasma (ICP) dry etching.^36^ Growth conditions for GaInN/GaN SLs and p-GaN shells of the samples are shown in Table 1, and detailed structures of samples a–l are illustrated in Fig. 1. The n-GaN cores of all samples were grown at 1150 °C for 78 seconds. To investigate the effect of SLs on the shape of NWs and emission properties, 20 pairs of GaInN/GaN SL structures were introduced in samples a, b, and c with different growth temperatures of 800, 780, and 760 °C, respectively. The sequential temperature gradient employed for growing the GaInN/GaN SL structures varied from the n-core to the MQS side. Specifically, the growth conditions for samples d, e, and f were as follows: 20 pairs at 800 °C, 10 pairs grown at 760 °C followed by 10 pairs grown at 800 °C, and 5 pairs grown at each of 760, 775, 785, and 800 °C, respectively. For samples g, h, and i, the growth temperatures of SLs followed a decreasing sequence: 20 pairs grown at 800 °C, 10 pairs grown at each of 800 °C and 760 °C, and 5 pairs grown at each of 800, 785, 775, and 760 °C, respectively. The growth times for the GaN barrier and GaInN wells in SL structures were 1.3 and 0.5 min, respectively, at the pressure of 90 kPa. There was no growth interruption when the SL growth temperature was varied. Afterwards, 5 pairs of MQS structures were coaxially grown in all the samples by using optimized growth sequences, as reported in our previous work.^31,37^ In samples j–p, the growth conditions of the SL were identical to that of sample a. The p-GaN shell on samples a–i and m–p was grown under the same conditions, including two stages at a constant growth temperature of 930 °C. A high growth rate step with a V/III ratio of 557 was performed for 3 min, followed by a Mg doping enhancement step with a V/III ratio of 1115 and grown for 30 s. The Mg concentrations of the first and second p-GaN shells were estimated to be 3 × 10^19^ cm^−3^ and 6 × 10^19^ cm^−3^, respectively.^38,39^ To investigate the effect of p-GaN thickness, different growth times of 3.0, 2.1, and 1.2 min were implemented for samples j, k, and l, respectively.

**Table 1:** NW samples with different growth conditions

Sample	Temperature and growth time for GaInN of the SL	Temperature and growth time for GaN of the SL	Pairs of GaInN/GaN SL	Temperature and growth time for the 1st p-GaN shell	Temperature and growth time for the 2nd p-GaN shell
a	800 °C, 1.3 min	800 °C, 0.5 min	20 pairs	930 °C, 3.0 min	930 °C, 0.5 min
b	780 °C, 1.3 min	780 °C, 0.5 min	20 pairs	930 °C, 3.0 min	930 °C, 0.5 min
c	760 °C, 1.3 min	760 °C, 0.5 min	20 pairs	930 °C, 3.0 min	930 °C, 0.5 min
d	800 °C, 1.3 min	800 °C, 0.5 min	20 pairs	930 °C, 3.0 min	930 °C, 0.5 min
e	760 °C, 1.3 min	760 °C, 0.5 min	10 pairs	930 °C, 3.0 min	930 °C, 0.5 min
	800 °C, 1.3 min	800 °C, 0.5 min	10 pairs		
f	760 °C, 1.3 min	760 °C, 0.5 min	5 pairs	930 °C, 3.0 min	930 °C, 0.5 min
	775 °C, 1.3 min	775 °C, 0.5 min	5 pairs		
	785 °C, 1.3 min	785 °C, 0.5 min	5 pairs		
	800 °C, 1.3 min	800 °C, 0.5 min	5 pairs		
g	800 °C, 1.3 min	800 °C, 0.5 min	20 pairs	930 °C, 3.0 min	930 °C, 0.5 min
h	800 °C, 1.3 min	800 °C, 0.5 min	10 pairs	930 °C, 3.0 min	930 °C, 0.5 min
	760 °C, 1.3 min	760 °C, 0.5 min	10 pairs		
i	800 °C, 1.3 min	800 °C, 0.5 min	5 pairs	930 °C, 3.0 min	930 °C, 0.5 min
	785 °C, 1.3 min	785 °C, 0.5 min	5 pairs		
	775 °C, 1.3 min	775 °C, 0.5 min	5 pairs		
	760 °C, 1.3 min	760 °C, 0.5 min	5 pairs		
j	800 °C, 1.3 min	800 °C, 0.5 min	20 pairs	930 °C, 3.0 min	930 °C, 0.5 min
k	800 °C, 1.3 min	800 °C, 0.5 min	20 pairs	930 °C, 2.1 min	930 °C, 0.5 min
l	800 °C, 1.3 min	800 °C, 0.5 min	20 pairs	930 °C, 1.2 min	930 °C, 0.5 min
m–p	800 °C, 1.3 min	800 °C, 0.5 min	20 pairs	930 °C, 3.0 min	930 °C, 0.5 min

**Fig. 2:**
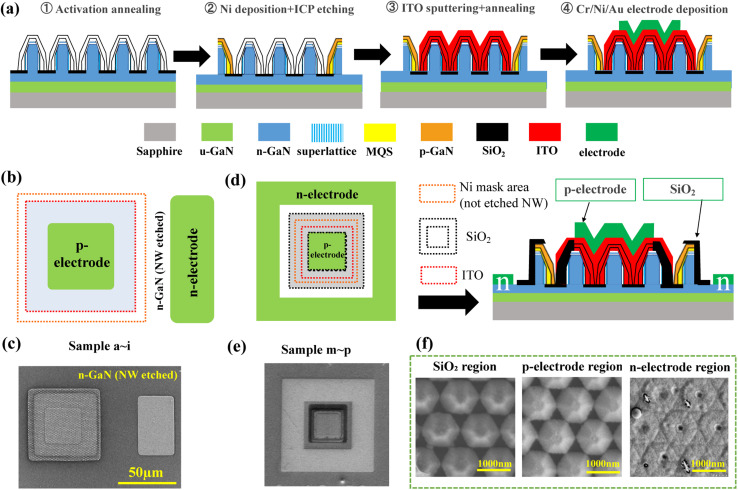
(a) Fabrication processes of the NW-based LED devices including ① activated annealing in a nitrogen atmosphere, ② deposition of Ni in the mesa area and removal of the NWs at the n-electrode area by ICP-based Cl_2_ etching, ③ indium tin oxide (ITO) sputtering on the mesa area followed by an annealing step, and ④ Cr/Ni/Au metallic film deposition on the electrodes. (b) Planar schematic structures and (c) SEM views of the 50 × 50 μm^2^ NW-LEDs in samples a–i. (d) Planar and cross-sectional schematics and (e) planar SEM views of NW-LEDs in samples m–p. (f) Planar SEM images of SiO_2_, p-electrode, and n-electrode regions.

Table 2 shows the emission area and detailed dimensions of the devices in the samples a–i and m–p. Fig. 2(a) schematically shows the device fabrication process for samples a–l. After epitaxial growth, thermal activation was performed at 650 °C for 30 min under an N_2_ atmosphere inside the MOCVD reactor to suppress oxide film formation on the p-GaN surface. Prior to the ICP etching of the n-electrode region, Ni thin film was selectively sputtered as the etching protection film in the mesa region using electron beam evaporation (EB, MA08-3065, ULVAC, Chigasaki, Kanagawa, Japan) with photolithography-based patterning. The indium tin oxide (ITO) transparent layer was deposited using a high-frequency magnetron sputtering system (CFS-4EP, Shibaura Mechatronics, Japan), followed by thermal annealing at 600 °C for 4 min in an N_2_ atmosphere. Finally, Cr/Ni/Au metallic films were sequentially deposited on the n- and p-electrodes with thicknesses of 10/20/200 nm using the EB evaporation. The devices were designed with a mesa size of 50 × 50 μm^2^ and a p-electrode area of 30 × 30 μm^2^, as shown in Fig. 2(b) and (c).

**Table 2:** Parameters of the device structures in samples a–l, m, n, o, and p

Sample name	a–l	m	n	o	p
SiO_2_ opening (μm^2^)	—	100 × 100	50 × 50	30 × 30	10 × 10
Emission area (μm^2^)	2500	10 000	2500	900	100
p-electrode area (μm^2^)	30 × 30	100 × 100	50 × 50	30 × 30	30 × 30
Ratio of the emission area to p-electrode area	0.36	1.0	1.0	1.0	3.0

To control the emission planes of NW-LEDs with different mesa areas, samples m–p were fabricated with a device structure different from that of samples a–l. The emission areas of samples m–p were 100 × 100, 50 × 50, 30 × 30, and 10 × 10 μm^2^, respectively. To eliminate the influence of the p-electrode area, the ratio of the p-electrode to the luminescence area is 1 for samples m, n, and o. The schematics of the device structures and the corresponding SEM images after fabrication are shown in Fig. 2(d) and (e). The emission areas of samples a–l were determined by the ITO area, whereas those of samples m–p were determined by the SiO_2_ opening. Therefore, the difference in the device fabrication process for samples m–p was that SiO_2_ sputtering on the edge of the mesa region was inserted between n-electrode etching and ITO sputtering. Fig. 2(f) shows the SEM morphology of SiO_2_, p- and n-electrodes, indicating that the SiO_2_ passivation layer and metal films were uniformly deposited in the specific regions. For optical characterization, *J*–*V*–*L* characteristics were measured using a semiconductor parameter analyzer (4156C, Agilent Technologies, Santa Clara, CA) and a mini-spectrometer (Hamamatsu Photonics, Japan). The following formula was applied to calculate the current density (*J*) of NW-LEDs:
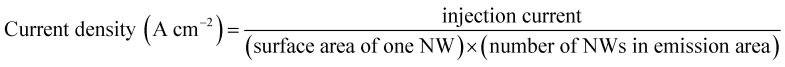


The surface area of one NW can be calculated from the cross-sectional SEM and the number of NWs can be estimated from the pitch of NWs and the emission area.^40^ Specifically, the EL signal was detected using a microprobe system with a fiber spectrometer (USB2000, Ocean Optics Inc., Largo, Florida). Both light output and EL spectra were detected from the backside of the sample on the wafer. Therefore, there is no effect of light being blocked by probes and p-electrodes.

## 3. Results and discussion

### 3.1 Effect of the growth temperature of the SL structure on the emission properties

**Fig. 3:**
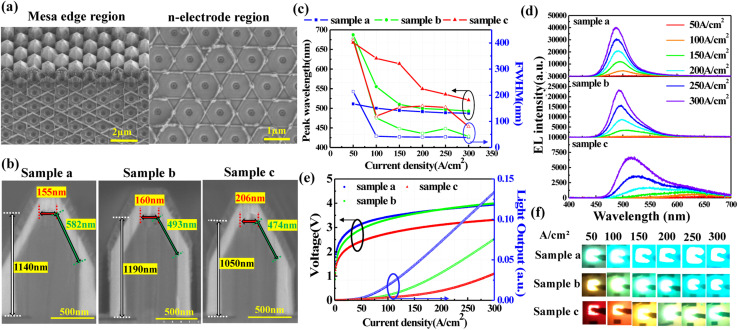
(a) SEM images of the mesa edge and the n-electrode area. (b) Cross-sectional SEM images of one NW in samples a, b, and c, respectively. (c) Emission peak and FWHM as a function of injected current density varying from 50 to 300 A cm^−2^. (d) EL spectra acquired at different current densities. (e) *J*–*V*–*L* characteristics as a function of current density. (f) Emission photographs of the samples captured at the injection current densities of 50, 100, 150, 200, 250, and 300 A cm^−2^.

The effect of the SL with different growth temperatures on the electro-optical properties of NW-LEDs was investigated by fabricating micro-devices on samples a, b, and c. Fig. 3(a) shows the SEM images of the etched surface after mesa part formation. The surface of the mesa edge region is rougher than that of the n-electrode region, because the NWs near the edge were not etched sufficiently. Although few residual unetched p-GaN shells exist because of the relatively slower etching rate, their impact on the Cr/Ni/Au metallic contact on n-electrodes and device performance is deemed negligible. The device fabrication processes for samples a, b, and c were performed simultaneously, and it has been confirmed that there was no clear difference in the device surface morphologies. Fig. 3(b) shows the cross-sectional SEM views of one NW in samples a, b, and c, respectively. As the SL growth temperature decreased, the (0001)-plane of the NWs became larger and the (11̄01)-plane shrunk. This is attributable to the lower growth rate on the (11̄01)-plane than on the (0001) and (101̄0)-planes, since the outermost surface of the (11̄01)-plane is composed of N atoms and easily passivated by hydrogen atoms.^31,41^ At a lower growth temperature, the thicker SL shell was grown on (11̄01)-planes, resulting in the larger (0001)-plane and smaller (11̄01)-plane. Consequently, the NW heights excluding p-GaN in samples a, b, and c were 1140, 1190, and 1050 nm, respectively. The variation in NW height mainly originated from the distinct thickness of the SL on the (0001)-plane at different growth temperatures.

EL measurements were performed to investigate the emission wavelength and emission plane of the NW-LEDs. Fig. 3(c) and (d) show the EL peak wavelength and full width at half maximum (FWHM) plotted as a function of current density and the EL spectra of samples a, b, and c, respectively. Since current density determines the position of the injectable current into the NWs,^42,43^ knee points are distinguishable in the peak wavelength curves, especially for sample c. The alteration in current density resulted in a more pronounced shift of peak wavelength for sample c with SLs growing at a low temperature, while it yielded a comparatively minor effect for the samples grown at higher temperatures. Specifically, the blue-shifts of the peak wavelength for samples a, b, and c were 13, 63, and 107 nm as the injection current density increased from 100 to 300 A cm^−2^. Since the top region of NWs usually manifests a higher InN mole fraction,^23,24^ the long wavelength emission was considered to be emitted from the (0001)-plane at the top of the NWs. For sample a, the (0001)-plane region was smaller than that of the other two samples, while the crystalline quality was also improved with a higher growth temperature of the SL. Therefore, the weak emission component from the (0001)-plane region was reduced, improving the stability of the emission peak in sample a. The decrease in FWHM with increasing SL growth temperature suggests that the degradation of MQS crystallinity in the (0001)-plane region is mitigated. In addition, the FWHM exhibited a decrease at each current density as the current density increased across all samples. This phenomenon is ascribed to the changed emission plane that occurs with the rising current density. Regarding the shape of the EL spectra, weak emission peaks located between 600 and 650 nm were observed in sample c. Although the (0001)-plane region showed potential to emit light with extended wavelengths, the low intensity is detrimental to the realization of high-efficiency NW-LEDs.

Fig. 3(e) and (f) show the *J*–*V*–*L* characteristics and corresponding emission photographs captured at different injection current densities. The threshold voltage of sample c was lower than that of samples a and b because of the longer emission wavelength in sample c at low current density. In all samples, the light output was rather small at current densities below 100 A cm^−2^ and rose with increasing current density. Such a phenomenon is attributable to the change in the emitting planes from the (0001)-plane to (11̄01)- and (101̄0)-planes of the NWs, since more current can be injected into the lower part of the NWs under the higher current density.^42,43^ In addition, the higher light output of sample a with SL grown at a higher temperature indicated that it is able to suppress current injection into the (0001)-plane. One can intuitively notice the increase in brightness and the alteration in the emission planes when examining the emission photographs, as shown in Fig. 3(f). Sample a showed a blue-green emission color at a current density of 50 A cm^−2^, and it can be confirmed that current was injected into the sidewall of the NWs. Similarly, samples b and c started to emit blue-green light from 100 and 200 A cm^−2^, respectively, confirming that the current was localized at the apex region under low current density. The decreased light output in the samples with a lower growth temperature of the SL was attributed to the injection current localized within the larger size of the (0001)-plane region. This phenomenon is associated with the low growth temperature of SL, facilitating the formation of a serpentine-shaped MQS structure on the (0001)-plane region.^37,39,41^ Lowering the growth temperature of the SL was intended to enhance the InN mole fraction of GaInN-wells in the SL for capturing point defects, but the low-temperature growth can induce a negative effect on the GaN-barrier in the SL.^44^ In general, this suggests that altering the growth temperatures of SLs and the accompanying modifications in NW shape yielded better enhancements in the emission characteristics of NW-LEDs, as compared to the effect of a high InN mole fraction in its role of suppressing point defect propagation to the MQS.

### 3.2 Investigation of the gradation of different growth temperatures of SL structures for micro-LEDs

**Fig. 4:**
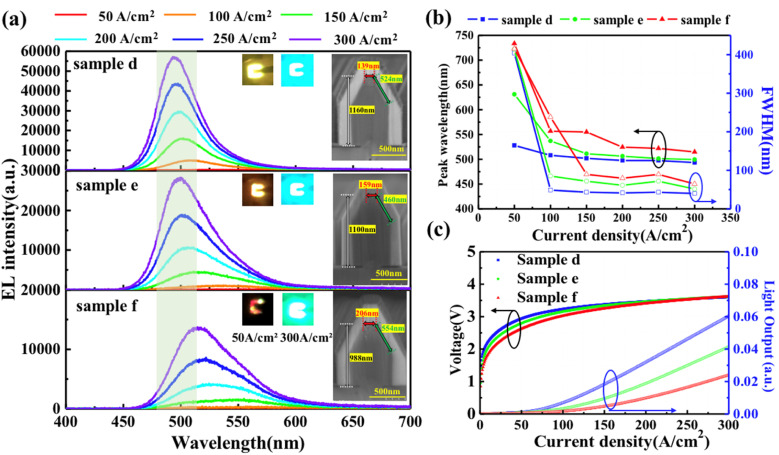
(a) EL spectra acquired at different current densities for samples d, e, and f. Insets show the cross-sectional view of SEM images of the NWs and emission photographs at current densities of 50 and 300 A m^−2^, respectively. (b) Emission peak and FWHM as a function of injection current density varying from 50 to 300 A m^−2^ (c) *J*–*V*–*L* characteristics of the samples d, e, and f.

The effect of the SL with gradation of different growth temperatures on the electro-optical properties of NW-LEDs was investigated. Samples d, e, and f were prepared with the growth temperatures of the SL escalating from 760 to 800 °C, while samples g, h, and i were grown using an opposite sequence from 800 to 760 °C. The samples with escalating growth temperature were intended to improve the surface flatness of the outermost SL layer on the apex region. Regarding the samples with decreasing growth temperature of SL, the aim was to increase the InN mole fraction in GaInN wells and to reduce the lattice mismatch with MQS structures. Fig. 4(a) shows the EL spectra measured in the fabricated micro-LEDs of samples d, e, and f at different current densities. The EL spectra of sample d with a constant growth temperature of the SL exhibited the highest intensity. In addition, as compared to samples d and e, the peak wavelengths in the marked green area were red shifted in sample f. This phenomenon is consistent with the color of the corresponding emission photographs captured at injection current densities of 50 and 300 A cm^−2^, as shown in the insets of Fig. 4(a). From the inserted cross-sectional SEM images, the (0001)-plane region for samples d, e, and f are 139, 159, and 206 nm, respectively. The correlation between emission wavelength and NW shape is also confirmed in Section 3.1, where the larger (0001)-plane area of NWs yielded a longer emission wavelength. The peak wavelength and FWHM of the EL spectra are replotted as a function of current density in Fig. 4(b). At the low injection current density of 50 A cm^−2^, the emission peaks of samples d, e, and f were 528, 631, and 733 nm, respectively. Beyond the current density of 100 A cm^−2^, the blue-shift of emission peaks was alleviated as a result of the injection of current from the apex to the sidewall of the NWs. The FWHM similarly exhibited a trend of rapid decrease, plateauing above 100 A cm^−2^. Fig. 4(c) shows the *J*–*V*–*L* characteristics of samples d, e, and f. The threshold voltages of samples e and f slightly decreased compared to that of sample d, which is related to the current localization at the (0001)-plane region. The variation trend of the light output for samples d, e, and f was consistent with that observed in the EL peak intensity. When comparing the light output of sample f at a current density of 300 A cm^−2^, those of samples d and e were enhanced by factors of 2.5 and 1.7, respectively. Thus, the emission properties of the samples with gradually increasing SL growth temperature were worse than those of the samples with constant SL growth temperature.

**Fig. 5:**
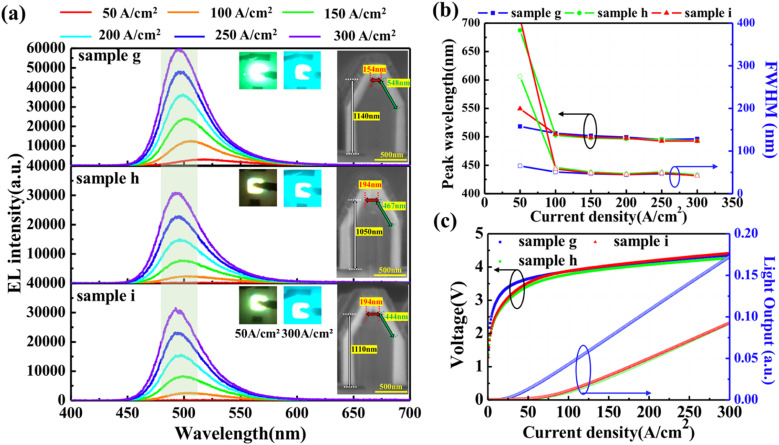
(a) EL spectra acquired at different current densities for samples g, h, and i. Insets show the cross-sectional view of SEM images of the NWs and emission photographs at current densities of 50 and 300 A m^−2^, respectively. (b) Emission peak and FWHM as a function of injected current density varying from 50 to 300 A m^−2^ (c) *J*–*V*–*L* characteristics of the samples g, h, and i.

Fig. 5(a) shows the EL spectra of the samples g, h, and i with gradually decreasing growth temperature of the SL. Beyond 100 A cm^−2^, the peak wavelengths of each sample exhibited negligible variances. The inserted cross-sectional SEM images indicate that the dimensions of the (0001)-plane are 154, 194, and 194 nm, respectively. The corresponding emission photographs were captured at current densities of 50 and 300 A cm^−2^. In Fig. 5(b), the peak wavelength and FWHM of the EL spectra are replotted as a function of current density to confirm the difference among the three samples. At the low current density of 50 A cm^−2^, the samples h and i with larger (0001)-plane regions tend to have longer peak wavelengths and larger FWHM. At high current densities, the emission peak wavelength and FWHM are same within samples g, h, and i. From the *J*–*V*–*L* characteristics in Fig. 5(c), the threshold voltage of sample g was slightly higher than the values of samples h and i. In addition, the light output was decreased in samples h and i with gradually decreased growth temperature of the SL. It indicated that the low emission efficiency of the (0001)-plane is still the dominant factor for light output, and the most effective way to realize high efficiency devices is to suppress current injection into the apex or minimize the grown (0001)-plane region. Based on the above NW-LED characteristics, it was confirmed that the size of the (0001)-plane region can be controlled to some extent by increasing the SL growth temperatures.

### 3.3 Effect of the thickness of the p-GaN shell on the emission plane control of NW-LEDs

**Fig. 6:**
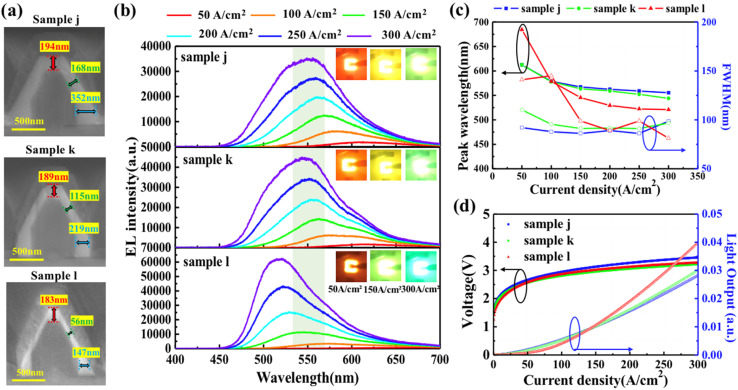
(a) Cross-sectional SEM images of samples j, k, and l. (b) EL spectra acquired at different current densities. Insets show the emission photographs at 50, 150 and 300 A cm^−2^. (c) Emission peak and FWHM as a function of injected current varying from 50 to 300 A cm^−2^ (d) *J*–*V*–*L* characteristics of the samples j, k, and l.

The (0001)-plane region was found to have a significant effect on the emission properties of the NW-LEDs. Therefore, we aimed to control the emission in the (0001)-plane region by changing the growth time of the p-GaN shell in samples j, k, and l, while the other conditions were identical. From the cross-sectional SEM view in Fig. 6(a), the thicknesses of the p-GaN shell on the (0001)-plane region of samples j, k, and l were 194, 189, and 183 nm, respectively. On the (11̄01)-planes, the p-GaN shell thickness was drastically decreased from 168 nm in sample j to 115 and 56 nm for samples k and l. The thicknesses of the p-GaN shell on the (101̄0)-plane were 352, 219, and 147 nm for samples j, k, and l, respectively. Therefore, the growth rate of the p-GaN shell was different on each plane of the NWs and with different growth times. In the early stage of growth, the growth rate of the (11̄01)-plane was smaller than that of the other planes. When the growth time was short in sample l, the p-GaN shell exhibited thicker growth on the (0001) and (101̄0)-planes and thinner growth on the (11̄01)-plane. Since p-GaN growth was accelerated in the lateral direction, the growth rate of the p-GaN shell on the (0001)-plane became small with increase in growth time, forming a large area of the (0001)-plane region of NWs in sample j.

**Fig. 7:**
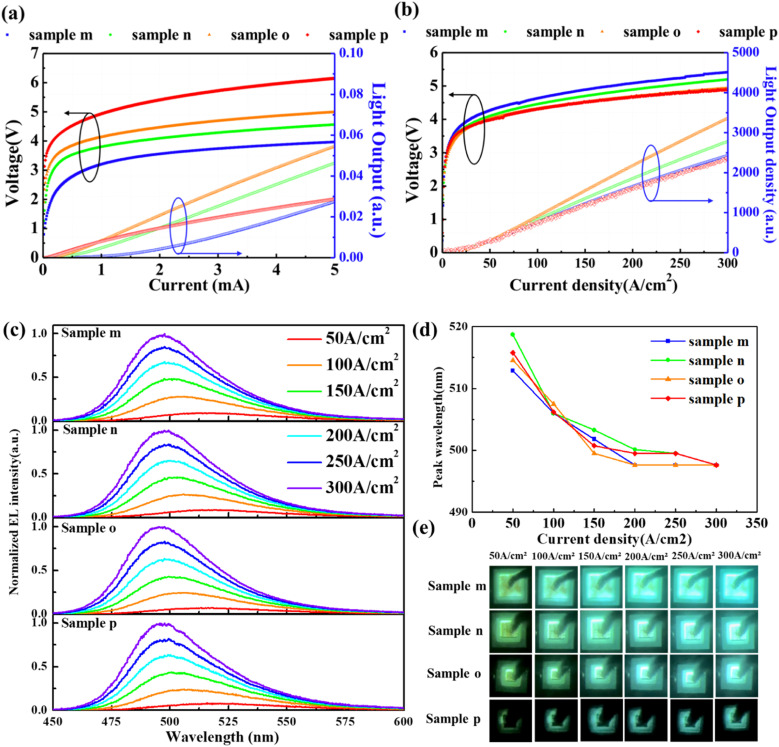
(a) *I*–*V*–*L* and (b) *J*–*V*–*L* characteristics of samples m, n, o, and p. (c) Normalized EL spectra acquired at different current densities. (d) Emission peak wavelength as a function of injected current density varying from 50 to 300 A cm^−2^. (e) Emission photographs at injection current densities of 50, 100, 150, 200, 250 and 300 A cm^−2^.

Fig. 6(b) shows the EL spectra acquired at different injection current densities. The broad EL spectra at 300 A cm^−2^ for each sample were Gaussian-fitted by triple peaks at 505, 545, and 590 nm, which were associated with different emission planes of the NWs. Insets show the emission photographs at 50, 150 and 300 A cm^−2^. Fig. 7(c) shows the plot of the EL peak wavelength and FWHM as a function of current densities. At the current injection density of 50 A cm^−2^, the peak wavelengths of samples j, k, and l were 612, 612, and 684 nm, and the FWHM of samples j, k, and l were 92, 110, and 141 nm, respectively. The short peak wavelength and small FWHM of sample j indicate that the emission from the (0001) plane region is suppressed in the low current density region for samples with longer p-GaN shell growth time. Compared to the p-GaN thickness on the (0001)-plane, the difference in the (11̄01)-plane region was pronounced, resulting in enhanced injection into the (11̄01)-plane of sample l at high current densities. At current densities below 150 A cm^−2^, the EL intensity was dominated by emission from the (0001)-plane of the NWs, resulting in a larger FWHM. This result suggests that a thin p-GaN shell on the (11̄01)-plane can promote current injection into the sidewall of the NWs, which shifted the peak wavelength to shorter wavelengths and reduced the FWHM. From the *J*–*V*–*L* characteristics in Fig. 7(d), there is no obvious difference of the operation voltage observed in the samples j, k, and l. Above the current density of 150 A cm^−2^, the light output of sample l surpasses that of samples j and k, owing to the varying thickness of the p-GaN shell and emission planes. Therefore, it indicates that the emission plane can be controlled to some extent by tunning the thickness of the p-GaN shell.

### 3.4 Effect of device size on the emission planes of NW-LEDs

Since the emission plane of NWs changed depending on the current density, it is necessary to further investigate the emission properties depending on the current density in terms of different device sizes. Different emission areas of 100 × 100, 50 × 50, 30 × 30, and 10 × 10 μm^2^ were designed and fabricated in samples m, n, o, and p, respectively. Fig. 7(a) and (b) show the *I*–*V*–*L* and corresponding *J*–*V*–*L* characteristics of samples m, n, and p as a function of injection current and current density, respectively. Due to variations in the emission area, the sample with a smaller size exhibited a higher current density when subjected to the same injection current. Therefore, the threshold and operation voltages of samples m, n, o, and p became smaller with an increase in device size of the samples, while such a variation showed the opposite trend as replotted in terms of current density. This result suggests that the contact resistance and current spreading through ITO may cause a higher operating voltage in samples with larger device sizes. Here, the observed higher operation voltages were induced by the specific structural design of the devices, which included additional SiO_2_ deposition on the edge region, in contrast to the other samples. Regarding the shape of the light output, the reason for the convex shape of sample m was associated with its large emission area and the small current density. At low injection current, the emission was dominated by the (0001)-plane region, and an increasing current was injected into the (11̄01) and (101̄0)-planes, resulting in the convex shape of the light output curve. For sample p with the smallest emission area, the concave shape of light output was attributable to the thermal saturation induced by the high current density. Normally, in (0001)-plane planar LEDs, the light output decreases with a reduced emission area. This trend was opposite in the case of NW-LED samples m, n, and o, which is attributable to the different emission planes as current densities changed. For sample p, the effective injection of current into the NW was restricted due to the larger size of the p-electrode compared to the emission region. Despite sample p in Fig. 7(b), the relationship between light output density and current density intuitively shows the improved emission by reducing the device size.

Fig. 7(c)–(e) show the normalized EL spectra, peak wavelengths as a function of current density, and the emission photograph captured at different current densities. The EL spectra of all the samples were normalized by the peak intensity of the emission curve with a current density of 300 A cm^−2^. The shape and peak wavelength of the EL spectra are quite similar for each sample. Specifically, the ratio of EL intensity at 550 nm and the peak wavelength is 0.045, 0.044, 0.034, and 0.044 for samples m, n, o, and p at the current density of 50 A cm^−2^. It indicates that the sample o promoted current injection into the sidewall and suppressed emission in the (0001)-plane region of the NWs as compared to the other samples. From Fig. 7(d), it can be observed that the shift in peak wavelength exhibited a similar trend across all the samples as the current density increased. Regardless of the device size, the emission peak shifted to shorter wavelengths as the current density increased. This suggests that the emission plane expanded from the (0001)-plane at apex to the (11̄01) and (101̄0)-plane regions, consistent with the aforementioned results. In Fig. 7(e), the luminescence photographs reveal a consistent emission color in samples m, n, o, and p when subjected to the identical injection current densities.

**Fig. 8:**
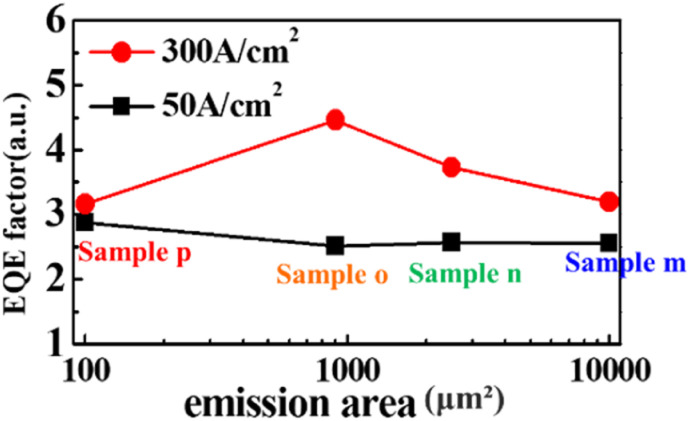
Estimated EQE factor as a function of the emission area of the NW-LED samples m, n, o, and p.

Since the light output of the LEDs was detected from the backside and cannot be directly converted into an absolute value, an approximate EQE factor was derived using the following equation:^45^
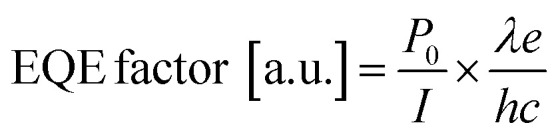
where *h* is the Planck constant, *e* is the elementary electric charge, and *c* is the speed of light. The values of the number of photons emitted per second (*P*_0_), the injection current (*I*), and peak wavelength (*λ*) were determined at the current density of 50 A cm^−2^ and 300 A cm^−2^, respectively. The luminescence properties were considered at 50 A cm^−2^, where luminescence in the (0001)-plane region is dominant, and at 300 A cm^−2^, where luminescence in the (11̄01)- and (101̄0)-planes is dominant. Fig. 8 shows the calculated EQE factors of NW-LEDs in samples m, n, o, and p with different emission areas. The corresponding parameters for EQE factor calculation were derived from the measurement results, as listed in Table 3. The EQE factor at 50 A cm^−2^ remained stable irrespective of the luminescence area. Typically, EQE tends to decrease as the emission area of the planar LED shrinks.^46^ This is because, as the LED size gets smaller, a larger proportion of the sidewalls contains high-density surface defects that originate from the mesa formation process. Conversely, in NW-LEDs, each NW in the mesa region operated independently, and surface defects mainly emerged at the p-GaN shell of the outermost NWs during the mesa process. Therefore, it is considered that the structural advantage of NW-LEDs suppresses the decrease in the EQE factor as the emission area drastically decreased. At the current injection density of 300 A cm^−2^, the EQE factor increased from sample m to o, while the value of sample p was equal to that at a current density of 50 A cm^−2^. The reason for the increased EQE factor of samples m, n, and o is attributable to the promoted current spreading to the sidewall of the NW under increased current density. Consequently, samples with smaller emission area exhibited superior current injection, resulting in more efficient light emission. For planar LEDs, the EQE factor decreased as the current density rose, primarily attributable to electron overflow and carrier localization.^47^ Conversely, in the case of NW-LEDs, the EQE factor increased as the current density increased, owing to the increasing predominance of emission from the (11̄01) and (101̄0)-plane. For sample p, the negligible variation of the EQE factor was assignable to the restricted effective injection due to the larger size of the p-electrode compared to the emission region.

**Table 3:** The parameters of current (*I*), wavelength (*λ*) and photons emitted per second (*P*_0_) for the EQE factor

	300 A cm^−2^			50 A cm^−2^		
	*I* (mA)	*λ* (nm)	*P*_0_ (mA)	*I* (mA)	*λ* (nm)	*P*_0_ (a.u.)
Sample m	33	498	0.032	5.5	513	0.27
Sample n	8.4	498	0.0085	1.4	519	0.078
Sample o	2.97	498	0.0030	0.50	515	0.055
Sample p	0.33	498	0.00038	0.055	516	0.0026

## 4. Conclusion

The optical and electrical properties of coaxial GaInN/GaN MQS NW-based LEDs were systematically investigated under varying growth conditions and device fabrication methods. Samples grown with higher temperatures of the SL demonstrated an enhanced growth rate along the [0001]-axis, leading to a reduced MQS size within the (0001)-plane region and an expansion of MQS in the (11̄01)-plane. This resulted in the suppression of the weak emission originating from the (0001)-plane region, ultimately enhancing light output and causing a blueshift in the peak wavelength. Afterward, NW-LED samples were fabricated by gradually changing the SL growth temperature, but the device maintained at a consistent growth temperature of 800 °C exhibited a greater light output. The results of the *J*–*V*–*L* characteristics indicated that the alteration in the NW shape resulting from high temperature growth played a more crucial role in the NW-LED performance than the reduction in the point defect propagation associated with low-temperature SL growth. To control the emission from the (0001)-plane region in NW-LEDs, a demonstration was conducted by using a thin p-GaN shell on the (11̄01)-plane. This led to enhanced current injection into the NW sidewall, resulting in a shift of the peak wavelength towards shorter wavelengths and an increase in the EL intensity. Furthermore, at the low injection current density of 50 A cm^−2^, the EQE factor of the NW-LEDs with different emission areas remained consistent. At the current injection density of 300 A cm^−2^, the EQE factor increased in the samples with a decrease in emission size. The reason for the increased EQE factor was ascribed to the promoted current spreading to the sidewall of the NW under increased current density. Overall, controlling the NW shape *via* inserting SLs and tuning current injection planes with different p-GaN shell thicknesses or emission areas are very important for the development of micro-LEDs.

## Author contributions

W. L. devised the experiments for this work. S. I. fabricated the LED devices, measured chip performance, and wrote the first draft of the manuscript. W. L. and A. S. grew the NW samples using MOVPE. W. L. and S. I. prepared all figures. W. L. analyzed the results, and revised the manuscript. W. L. and S. I. prepared the response to reviewers' comments. S. I., Y. H., and K. K. contributed to the device fabrication and characterizations. M. T. and Y. Y. supported the epitaxial growth and joined the discussion. K. H. joined the discussion. S. K. joined the discussion and revised the manuscript. S. K., T. T., and M. I. contributed to the data analysis and supervised the project.

## Conflicts of interest

There are no conflicts to declare.

## Supplementary Material
